# Identification of Metabolites from *Catharanthus roseus* Leaves and Stem Extract, and In Vitro and In Silico Antibacterial Activity against Food Pathogens

**DOI:** 10.3390/ph17040450

**Published:** 2024-03-30

**Authors:** Qazi Mohammad Sajid Jamal, Varish Ahmad

**Affiliations:** 1 Department of Health Informatics, College of Applied Medical Sciences, Qassim University, Buraydah 51452, Saudi Arabia; 2 Health Information Technology Department, The Applied College, King Abdulaziz University, Jeddah 21589, Saudi Arabia; vaahmad@kau.edu.sa

**Keywords:** *Catharanthus roseus*, NMR, indole alkaloids, secondary metabolites, antibacterial, food preservation

## Abstract

The plant produced powerful secondary metabolites and showed strong antibacterial activities against food-spoiling bacterial pathogens. The present study aimed to evaluate antibacterial activities and to identify metabolites from the leaves and stems of *Catharanthus roseus* using NMR spectroscopy. The major metabolites likely to be observed in aqueous extraction were 2,3-butanediol, quinic acids, vindoline, chlorogenic acids, vindolinine, secologanin, and quercetin in the leaf and stem of the *Catharanthus roseus*. The aqueous extracts from the leaves and stems of this plant have been observed to be most effective against food spoilage bacterial strains, followed by methanol and hexane. However, leaf extract was observed to be most significant in terms of the content and potency of metabolites. The minimum inhibitory concentration (20 µg/mL) and bactericidal concentrations (35 g/mL) of leaf extract were observed to be significant as compared to the ampicillin. Molecular docking showed that chlorogenic acid and vindolinine strongly interacted with the bacterial penicillin-binding protein. The docking energies of chlorogenic acid and vindolinine also indicated that these could be used as food preservatives. Therefore, the observed metabolite could be utilized as a potent antibacterial compound for food preservation or to treat their illness, and further research is needed to perform.

## 1. Introduction

A foodborne infection’s symptoms can range from completely asymptomatic to fatal. In the US, foodborne diseases cause 48 million episodes of sickness, 128,000 admitted to hospitals, and 3000 fatalities annually [1]. Proper handling and usage of preservatives have reduced microbial growth and economic loss of food. Infections caused by drug-resistant microorganisms, such as those in water and food, also pose a significant risk to public health [2]. Biosensors have been used to detect, identify, and manage microbial contamination in agricultural and food products for disease prevention and outbreak analysis [3,4]. Metal nanoparticles have the potential to kill numerous drug-resistant bacteria, including those in the Gram-positive and Gram-negative groups [3,4]. Although inorganic nanoparticles have several biocidal capabilities, the method by which they exert their powerful antibacterial efficacy is not as well understood. However, it was thought that releasing ions into solution produces reactive oxygen species that harm bacteria. As other studies show, nanoparticles are so small that they can pass through bacterial cell walls and target their organelles. The inorganic analogs of organic antibiotics are more effective against drug-resistant bacteria because they use many mechanisms of action [5,6]. Inorganic metal oxide nanoparticles, such as the nano-complex of metal oxide, are well admired for their bacteriostatic and bactericidal effects [7].

Similarly, various plants have been described as having significant antimicrobial potential against various pathogens [8]. Recognition and signaling caused by transcription components, alterations in hormone levels, and the creation of secondary metabolites are only a few examples of the many mechanisms involved in stressful reduction reactions. Particularly intriguing are secondary metabolites due to their roles in controlling plant interactions with their environments and subsequent adaptive responses. In a variety of environmental conditions like the lack of moisture and heat, plants increase their production of phenylpropanoid metabolic products like scopolamine, sinapic acid, sinapoyl aldehyde, and flavonoids, all of which support the generation of significant structural substances like lignin while also in reducing oxidative damage and preventing photoinhibition [8,9]. Establishing an equilibrium between the production of the natural therapeutic compounds of attention and the production of the various distress metabolites required by the herb to endure the stress is crucial to the efficacy of the significant molecules [10].

It is widely acknowledged that the *Catharanthus roseus* (L.) *G. Don* (*C. roseus*), or Madagascar periwinkle, is a member of the family *Apocynaceae*, used as a natural remedy in various cultures. For several decades, research and therapy have concentrated on the bioactive compound’s potential to be employed as an anticancer as well as an antimicrobial [11]. The plant has also been described for its many pharmacological activities. However, the extraction of bioactive compounds also generates solvent-mediated air, water, and soil pollution. The increase in toxic concentrations in the environment endangers human health, diminishes ecological functioning, and decreases wildlife populations [12]. However, extraction is an important step in the search for phytochemical bioactive molecules and translating their potential into significant therapeutic molecules.

There was also a discussion of more current developments in the application of cell, tissue, or callus cultures to produce desirable phytochemicals from various plants, including *C. roseus* [13,14]. Medications used in chemotherapy are a major part of clinical oncology evaluations. When taken orally, most of these drugs kill tumor cells but have the potential to harm healthy cells as well. These negative effects restrict the use of chemotherapy medications despite their high effectiveness in killing cancer cells.

Therefore, searching for complementary or alternative drugs that would be selectively effective with no adverse effects on health is an active area of research. A lot of these studies are looking into traditional herbal remedies used by different cultures around the world. Moreover, a report from the WHO [15] stated that nearly 80% of the global population relies on medications derived from plants, either entirely or partially. Antimicrobials, cancer, diabetes, fever, hemostasis, and hypertension are only some of the conditions that have been treated using the plant *C. roseus* during traditional medical practices in a number of different societies [16,17]. However, few reports have addressed the effects of *C. roseus* crude aqueous extract in the field of food preservation. This study is aimed to identify the possible primary and secondary metabolites of leaves and stem of *C. roseus* using NMR spectroscopy. We also analyzed the antibacterial potential of different solvents and explored the possible molecular interaction of most interacting molecules with bacterial targets.

## 2. Results and Discussion

**Figure 1:**
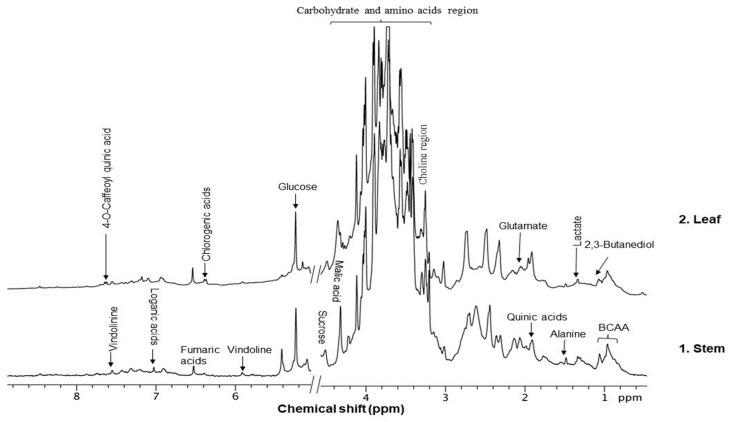
Typical 1 H-NMR spectra of the metabolites of the different sections of *C. roseus*: (1) leaves and (2) stems. Key metabolites: Branch chain amino acids (BCAA), 2,3 butanediol, quinic acids, vindoline, lactate, alanine, pyruvate, glutamate, malic acid, glucose, vindoline, chlorogenic acids, fumaric acid, vindolinine, secologanin, quercetin, respectively.

The collected plant leaves and stems were used to prepare their extractions in water, methanol, and hexane. Each extract was checked for its antibacterial activities; leaves and stem extractions with water were observed to be the most significant fraction for antibacterial activities. Therefore, the aqueous fraction underwent NMR analysis to determine the presence of potential metabolites. The analyzed sample fraction peak and its metabolites are shown in Figure 1.

Metabolite fingerprinting, profiling, and footprinting are the components of methods used for metabolomics studies. Profiling helps to identify and characterize metabolites of a specific metabolic pathway. The main goal of fingerprinting is to compare and distinguish between samples using high-throughput screening and extraction of the metabolite content of different sections of *C. roseus*, like the leaf and stem. Footprinting is the fingerprinting analysis of identified metabolites, which were assigned with the help of NMR one-dimensional spectra of leaf and stem samples. Unlike COSY (homonuclear correlation spectroscopy), which only generates connections between vicinal or geminal protons, TOCSY (Total Correlation Spectroscopy) generates correlations among all protons in a specific spin system. 2D NMR-based methods, mainly TOCSY and COSY, are commonly used for molecule recognition in plant extracts to assign peaks to metabolites accurately. After the identification of the metabolite, a characterization analysis of the NMR spectra assignment of the aliphatic, aromatic, and sugar regions was performed to find discriminating signals in the leaf and stem. They offer an outstanding platform for studying., for example, the anticancer and other medical chemicals, quality control as well as authentication of medicinal plants, their classification at the level of species ecotypes, genotypes, identification of biomarkers for disease diagnosis, and therapeutics. Metabolites including branch chain amino acids (BCAA), 2,3 butanediol, quinic acids, vindoline, lactate, alanine, pyruvate, glutamate, malic acid, glucose, chlorogenic acids, fumaric acid, indoline, secologanin, and quercetin.

### 2.1. Assessment of the Antimicrobial Impact

**Figure 2:**
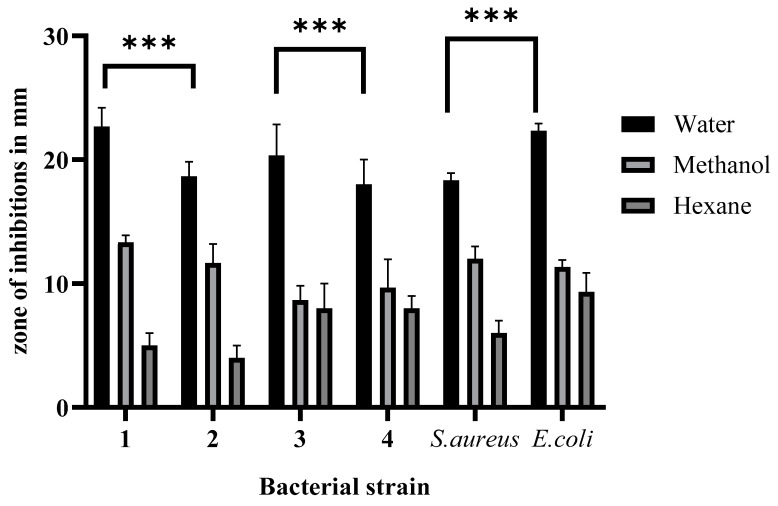
Antibacterial effect of water, methanol, and hexane extracts of *C. roseus* leaves against meat-isolated bacterial pathogens (1, 3 Gram-negative *streptobacilli* and 2, 4 Gram-positive *Streptococcus*). The significant values were expressed as *** *p* value 0.0001.

The aqueous, methanolic, and hexane extracts were used to analyze the antibacterial effect by agar well diffusion assay against Gram-positive isolates presented as 2, 4, and *S. aureus* and Gram-negative bacteria 1, 3, and *E. coli*. Ampicillin was used as a standard drug. The observed results and the effect of solvent are shown in Figure 2.

The aqueous extracts of leaves have a stronger antimicrobial effect on Gram-negative bacteria than on Gram-positive bacterial isolates, which were subsequently followed by methanol and hexane. Similarly, Gram-negative bacteria are potentially inhibited compared to Gram-positive reference bacteria. Nutrient broth medium was solely utilized as a negative control; Ampicillin was a positive control. *S. aureus* was used as a Gram-positive reference strain, and *E. coli* was used as a Gram-negative reference bacterium strain (Figure 2).

**Figure 3:**
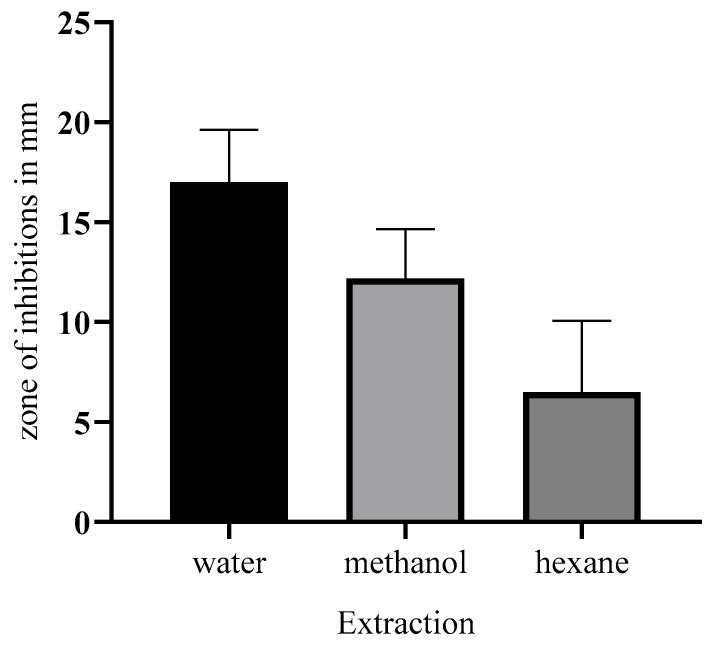
Solvent effect on extraction of metabolites and antimicrobial activity of leaf extract of *C. roseus.* The hexane extract was likely observed to have the least growth inhibitory effect on the bacterium in a medium with methanol, while the optimum extraction had a strong growth inhibitory effect on the bacterium.

Gram-positive and Gram-negative bacteria isolated from spoiled chicken meat were subjected to the antibacterial effect test. The aqueous extract of leaves has shown stronger inhibition against Gram-negative (22 mm zone of inhibition) bacterial isolates than Gram-positive bacteria. The growth of the standard test indicator *E. coli* has been observed to be inhibited more strongly than that of *S. aureus* (18 mm zone of inhibition). The minimum inhibitory concentrations were 12.5 and 20 g/mL, respectively. The minimum bactericidal concentrations for Gram-negative and Gram-positive bacteria isolates were 20 and 35 g/mL, respectively. The antibacterial effects of stem extract were also analyzed, and hexane and methanol (13 mm zone of inhibition), and hexane (8 mm zone of inhibition) were found to be less significant in comparison to the leaf aqueous extract; the antibacterial effect and significance of extracting solvent are shown in Figure 2 and Figure 3. Significant results were also obtained regarding the stem extract’s inhibitory impact and strength against Gram-negative bacteria, but it was less than that of the leaves of the plant.

### 2.2. Virtual Screening and Docking Results

**Figure 4:**
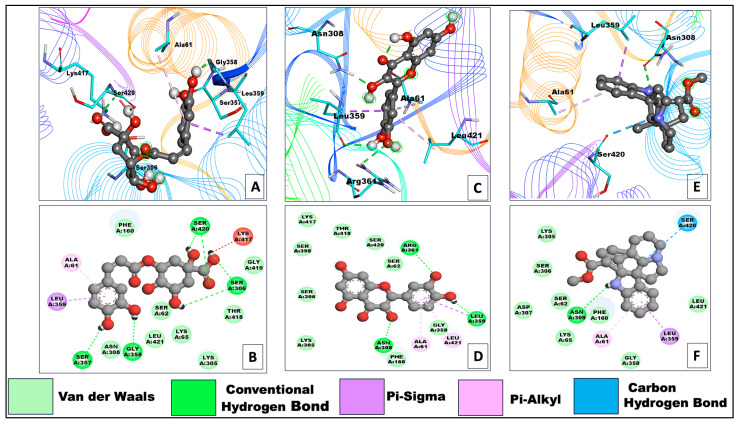
Showing 2D and 3D representations of best-screened compounds and their interaction against penicillin-binding protein 4 (dacB) from *Escherichia coli* (PDB:2EXB). (**A**,**B**) represents 2exb-chlorogenic Acid, (**C**,**D**) represents 2exb-quercetin, and (**E**,**F**) represents 2exb-vindolinine complexes. The receptor molecules (PDB:2EXB) are shown by a rainbow line ribbon pattern, and compounds are in the center, shown by a grey ball and stick shape. In the 3D representation, a cyan stick pattern indicates the most interacting amino acids. In 2D representation, the multicolor spheres show amino acid residues forming different types of interactions.

The interactions with bacterial receptors were screened via docking studies. The docking scores suggested that the 2exb-Chlorogenic Acid complex showed the best binding energy, i.e., −7.7 kcal/mol, which was supported by six hydrogen bonds (Table 1), which was more than the used control drug (complex: 2exb-Flomoxef) binding energy −7.1 kcal/mol. The interactions through which Chlorogenic Acid could mediate its antibacterial effect are represented in Figure 4A,B (Table 1). Many phenolic molecules have been described as having potential antibacterial and pharmacological activities. Chlorogenic acid (CA), as analyzed in this study by NMR, is a phenolic compound that could be responsible for antibacterial and antibiofilm activities. The high antibacterial efficacy and antibiofilm assays against *S. maltophilia* in both in vitro and in vivo were also examined (MIC > 32 g mL^1^), including the TMP/SMX-resistant strain. Chlorogenic acid and plant products containing chlorogenic acid have demonstrated potent antifungal, antibacterial, and antiviral properties. It has been documented that some bacterial pathogens inhibit both Gram-positive (*Streptococcus pneumoniae*, *Streptococcus mutans*, *Staphylococcus aureus*, *Bacillus subtilis*, and others) and Gram-negative (*Shigella dysenteriae*, *Salmonella typhimurium*, and *E. coli*) [18].

The antibacterial effect of chlorogenic acid has been described against *A. Acidoterrestris* [19] and *Yersinia enterocolitica*. Drugs that target penicillin-binding proteins may inhibit the spread of disease-causing bacteria. Chlorogenic acid destroys bacterial cells, as reported earlier, lending credence to the findings of this investigation. A second compound, 2exb-quercetin, with a docking energy of −7.5 kcal/mol and the formation of two hydrogen bonds, performed similarly well (Figure 4C,D and Table 1). Quercetin is a common flavonoid with antibacterial properties. Researchers have investigated the bacteriostatic effect of quercetin on *E. coli*, *Salmonella enterica*, *P. aeruginosa*, *S. aureus*, and *S typhimurium*, as well as the antibacterial mechanism of quercetin in *S. aureus* and *E. coli*. Quercetin had a more pronounced effect on Gram-positive bacteria than Gram-negative bacteria as a bacteriostatic. *E. coli* (at 50 MIC) and *S. aureus* (at 10 MIC) suffered membrane and wall damage when exposed to quercetin. The other screened molecule was vindolinine, which was shown to interact more strongly but less than chlorogenic acid. Complex 2exb-indoline has been observed to show very near and less binding energy, i.e., −6.9 kcal/mol, than control drug binding energy, and interaction with the bacterial receptor could be mediated through the involvement of two hydrogen bonds (Figure 4E,F and Table 1). It was observed that amino acid residues mainly SER62, ARG361, SER420, GLN422, SER357, SER306, LEU421, ASP155, GLY358, SER306, ASN308, ARG361, and LEU359 were involved in the formation hydrogen bonds during 2exb interaction with selected compounds (Table 1). Meanwhile, amino acid residues ALA61 were found in hydrophobic interaction in all complexes (Table 1).

The binding and interaction patterns of all selected complexes are depicted in the following Figure 4. 3D and 2D graphics have been developed for visual interpretation.

The herbal remedy from *C. roseus* is found in tropical regions. Antibacterial activity in the plant’s leaves has been demonstrated against various microorganisms, notably *S. aureus, E. coli*, and *C. albicans.* Catharanthine, Vincristine, and vinblastine are only some of the alkaloids found in *C. roseus* leaves, and they’re responsible for the plant’s antibacterial properties [20]. These alkaloids have been reported to kill bacteria by interfering with their reproduction ability. Moreover, there are phenolic chemicals in *C. roseus*, which have antibacterial effects [21,22].

These chemicals are believed to kill bacteria by causing membrane disruption within their cells. Several bacteria, like *S. aureus, E. coli,* and *P. aeruginosa,* were killed by an ethanolic leaf extract. The alcohol-based extract of the leaves was used to treat *S aureus*-induced infection of the skin in rats, as described in vivo research. The medication successfully mitigated the disease’s effects [22]. Antibiotic agents, in combination, provided combined benefits for *Candida albicans* who have developed fluconazole resistance. Antimicrobial activities against *E. coli*, *S. aureus*, MRSA strains, and *C. albicans* have also been detected with the ethanol extract of *C. roseus* leaves. Although the precise mechanism(s) by which *C. roseus* inhibited bacterial growth remains unknown, it is likely a combination of several factors. Among these are the alkaloid compounds in *C. roseus*, which can cause the bacterial cell membrane to become permeable and allow vital chemicals to escape. The alkaloids generate free radicals that can cause DNA damage, which stops bacteria from multiplying and interferes with the protein synthesis of bacteria [23]. Studies have shown that the antibacterial effects of *C. roseus* leaves can be attributed to their phenolic and alkaloid content. This plant’s ideal dosage and administration method have yet to be determined, and more research is required to establish its efficacy in treating bacterial infections. Being a sensitive analytical technique, NMR is used in the identification of metabolites derived from plants, animals, and microbes.

Fingerprinting circumvents the labeling difficulty caused by the abundance of signals in high-resolution 1H NMR spectra by comparing multiple spectra and, by extension, the specimens that were used when these spectra were produced via the application of multivariate analysis. Metabolites in the living things that were subjected to NMR analysis are the sources of the signals, as seen in the spectra. Finding out whether a collection of samples has similar or dissimilar spectra is more important than determining which metabolites are present. Originally created for use in healthcare applications, this type of pattern detection for analyzing NMR spectra has lately found its way into the examination of plant-based extracts and materials [24].

The phenolic found in plants encompass various categories of chemicals, including simple phenolic, flavonoids, phenolic acids, flavonoids, lignins, and tannins; molecules are characterized by the presence of a minimum one aromatic ring with at least one hydroxyl group attached. The hydroxyl group can either be unbound or involved in another chemical function, such as ether, ester, or glycoside. They are commonly found in plants and are often found in higher concentrations, either as soluble or cell wall-bound chemicals, due to the plant’s relation to the atmosphere.

Some primary metabolites like branch chain amino acids (BCAA), lactate, alanine, pyruvate, glutamate, malic acid, glucose, and fumaric acid were likely to be found in *C. roseus* in variable amounts in different parts of the stem and leaf of the plant as we also studied using NMR spectroscopy. Furthermore, some important peaks of secondary metabolites were most likely found in NMR spectra of the leaves aqueous extract of leaves, like 2,3 butanediol, quinic acids, vindoline, chlorogenic acids, vindolinine, secologanin, and quercetin. These metabolites dominated intensity and were detected by NMR in a mixture of plant tissue.

Monoterpenoid Indole Alkaloids, also known as MIAs, are a fascinating class of specialized metabolites that possess a vast array of chemical structures. They are also a source of a number of active biomolecules, including significant pharmacophores like anticancer drugs, camptothecins, and Vinca alkaloids, which are based on this type of structural configuration. The latter substances are found in low concentrations in the leaves of the *C. roseus*. They are the product of a complicated metabolic process, which is the focus of increasing research efforts [25].

The MICAs originate from a one-of-a-kind polyvalent framework that is referred to as strictosamide. This important precursor is the condensed product of an amine derived from tryptophan that has been coupled to a monoterpenoid moiety that has undergone substantial modification. While tryptophan decarboxylase (TDC) facilitates a solitary reaction wherein tryptophan is converted to tryptamine, the organization of the monoterpene secoiridoid moiety needs many reactions to produce secologanin using methyl-erythritol phosphate (MEP) pathway-derived monoterpenoid skeleton [12,26]. Four genes have been identified as being involved in the synthesis of vindoline, a terpenoid indole alkaloid that is an essential component of *C. roseus* leaves. Nevertheless, the information regarding the spatial arrangement of the tabersonine-to-vindoline biosynthetic route still needs to be completed. Previous studies were able to prove the existence of transcripts of the enzyme 16-hydroxytabersonine 16-O-methyltransferase (16OMT), which are specifically localized to the aerial organ epidermis, by using in situ hybridization [27,28]. Acids are a significant group of plant compounds that serve as a crucial layer of defense on leaves to defend against both living and non-living threats. Phenolic chlorogenic acids are prevalent in several plants and exhibit significant antibacterial characteristics. Investigations have shown that chlorogenic acid successfully hinders the growth of many bacteria, including *Shigella dysenteria* and *S. pneumonia* [29], and it was effective against both Gram-negative and Gram-positive bacteria. Furthermore, the coffee bean contains unusually high concentrations of chlorogenic acids, which are extensively dispersed metabolites in plants. The main chemical structure is a conjugate of caffeic acid (3,4-dihydroxy cinnamic acid) and tetrahydroxy-cyclohexane carboxylic acid (quinic acid). Quercetin is the primary phenolic component of this plant and exhibits antimicrobial properties. The antibacterial activity observed in this study could be due to the presence of quercetin, which needs to be explored more in the future [30]. Hydroxylated phenolic molecules with an aromatic ring structure are known as flavonoids. We observed the presence of quercetin, which belongs to this group of chemicals. It is a naturally occurring bioactive chemical with a flavonoid configuration nC6(ring A)-C3(ring C)–C6(ring B). Numerous physiological activities in plants, including pollen proliferation, plant growth, seed germination, photosynthesis, and the formation of antioxidant machinery that confers tolerance against both abiotic and biotic stresses, are all facilitated by quercetin. Consequently, this plant’s quercetin has a strong protective effect that has to be investigated further through in vivo research [31,32].

NMR analysis is a useful technique for metabolite analysis despite being somewhat limited by its degree of sensitivity. These analyses can enhance high-throughput system-wide investigations by MS. Utilizing coupled techniques that enable parallel MS and NMR investigations on the same sample is an effective method to expand the portion of the metabolites that can be uncovered through routine study. The latest development in metabolomics has been caused by the need for more in-depth analyses to support the discovery of the metabolic process network and the potential use of metabolomics to reveal gene function. The technique involves utilizing metabolite data to uncover the phenotype of silent mutations by analyzing metabolite signatures by 1H-NMR analysis. Nuclear magnetic resonance spectroscopy (NMR) is commonly used independently to characterize and identify metabolomics samples despite their inherent complementarity. The ability to compare NMR data with references or to use two-dimensional NMR to explain structure is the primary advantage of NMR metabolomic research [33]. This study allows for the investigation of primary metabolites, which include organic acids, sugars, and amino acids, as well as secondary metabolites, which include phenolic compounds, which are frequently abundant in plants. Metabolites such as 2,3 butanediol, quinic acids, vindoline, chlorogenic acids, vindolinine, secologanin, and quercetin in plants extracted from *C. roseus* leaf and stem used as drugs for anticancer other medications used for human health and treatment system. The aerial parts of the plants have shown significant antibacterial activities against bacterial pathogens.

## 3. Material and Methods

### 3.1. Plant Material

We collected the cultivated plant material to make 100 g of powder in the months of February and March from the NCR, Ghaziabad, at 28.6692° N and 77.4538° E. At the CSIR-National Botanical Research Institute in Lucknow, India, Dr. K.M. Prabhu Kumar authenticated the cultivated plant (PDSH/LWG/Authentication/Ang./2023-24/47) as *C. roseus* and subsequently deposited it at the same institute under the accession number 119224. The aerial sections of *C. roseus*, such as the leaf and stem, were selected for extraction, in vitro antibacterial activities, and metabolite identification and characterization using NMR spectroscopy. The selected leaves and stems of the plants were collected, kept in the shadows for drying, and powdered. Extraction was carried out at room temperature on a shaker using 10 g/100 mL of water, methanol, and hexane as a solvent in different flasks and used for antimicrobial activities.

### 3.2. Preparation of Extract

Fresh plant materials were used for metabolite identification. We transferred the fresh and sterilized tubes to the container holding the liquid nitrogen. Frozen tissue was stored for a few hours at −80 °C before drying. However, metabolites may degrade while being stored. The frozen plant parts were ground using a pre-cooled pestle mortar and liquid nitrogen. Transfer the ground leaf and stem into a plastic tube using a spatula. Subsequently, it was mixed and homogenized using the vortex for one minute at 25 °C. Furthermore, 1 to 2 min of room-temperature ultrasonication was performed. After ultracentrifugation, the extract was divided into 2 mL Eppendorf tubes and spun at 4 °C at 10,000 rpm for 10 min. To achieve a clear supernatant, the debris was separated. More time is needed to obtain a clean supernatant with higher-speed centrifugation. More than 1 mL of clear extract was transferred to a 1.5 mL fresh tube. Centrifugation (10,000 rpm, at 4 °C, for 10 min) was again repeated to achieve more clear supernatant and lyophilized. The term “lyophilization” refers to the technique of removing water from frozen products by placing them in a vacuum, resulting in the ice melting and turning into vapor without ever touching a liquid state. Furthermore, 1 to 2 min of room-temperature ultrasonication was also operated.

In preparation for additional NMR analysis, the sample was lyophilized, dried, powdered, and kept at −80 °C. The frozen powder was transferred into Eppendorf tubes. After dissolving the dry powder in 550 µL of D_2_O, it was moved to a 5 mm NMR tube. 0.75 mL of potassium dihydrogen phosphate buffer (KH_2_ PO_4_) in D_2_O was added to the tube containing a mixture of the plant sample and 550 µL of D_2_O [34,35].

**Figure 5:**
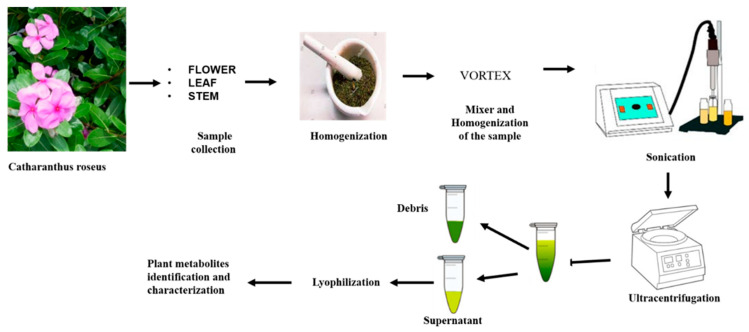
Simple presentation of sample collection, preparation, and metabolite extraction from *Catharanthus roseus* from different parts like flower, leaf, and stem.

An overview of sample collection, preparation, and crude extraction of leaf and stem from *C. roseus* is given in Figure 5.

### 3.3. Determination of Antibacterial Activity

To evaluate the antimicrobial significance of the leaves and stem extracts, each extract’s antibacterial activity was tested against reference strains and both Gram-positive and Gram-negative meat-derived bacterial isolates. For isolation, the meat sample was suspended in sterile water and kept in open air at room temperature for 2 days for the growth of microbes. The sample was inoculated on a solidified agar medium, grown at 37 °C overnight. From the grown colonies, two Gram-negative and two Gram-positive bacterial isolates were identified by Gram staining and used for a more comprehensive evaluation of the plant extracts’ antibacterial properties. The reference bacterial strains were Gram-negative, *E. coli* (ATCC 9637)*,* and Gram-positive, *S. aureus* (ATCC3538). Using agar well diffusion, extracts from the leaves and stems were evaluated for their capacity to regulate the growth of the bacterial indicators [36]. Agar plates were seeded with a bacterial culture that had grown overnight to find the minimal inhibitory concentration and the minimum bactericidal concentration. Antibiotic ampicillin (20 µg) was employed as a benchmark medication to measure the relative antibacterial efficacy of each extract. The statistics were run on GraphPad Prism 9.4.0 (San Diego, CA, USA). The antibacterial activities were analyzed three times. Therefore, the results are the average Standard Deviations for *n* = 3 sample size. A one-way analysis of variance (ANOVA) was used to determine the statistical significance. The *p*-value was used to evaluate the level of significance. If *p* is less than 0.05, the result is regarded as significant. The significant values were expressed as *** *p* value 0.0001.

### 3.4. Virtual Screening and Docking Studies

The virtual screening was performed with the chemical components of the extracted fraction that showed potent antibacterial activities (water) to find the most interacting molecules with the bacterial drug target (Table 2) directly obtained from the PubChem database [37]. From their SMILS IDs, the 3D structures were generated using the NovoPro online server (https://novoprolabs.com/tools/smiles2pdb) (accessed on 25 December 2023), and 2D structures were generated by Smi2Depict online tool (accessed on 22 February 2024) [38]. Later, the bacterial target crystal structural configuration of penicillin-binding protein 4 (dacB) from *Escherichia coli* (PDB:2EXB) [39] was downloaded from Protein Data Bank (PDB) [40] for molecular docking purposes, and interaction was performed by PyRx tool version 0.8 [41] and AutoDock 4.0 modules as adapted by Jamal et al., 2023 [42,43]. The energy minimization, 3D conformation analysis, and visualization of complexes were performed by Discovery Studio Visualizer 2021 v. 21.1.0.20298 [44] https://discover.3ds.com/discovery-studio-visualizer-download (accessed on 31 December 2023).

The most likely metabolites observed to be in the aqueous fraction of leaves and stems of the C. roseus plant were 2,3-Butanediol, Quinic Acid, Vindoline, Malic Acid, Chlorogenic Acid, Fumaric Acid, Vindolinine, Secologanin, and Quercetin, which were used in vertical screening. The dominant components of the aqueous extract of leaves are 2,3 butanediol, 4-0 caffeoyl, quinic acids, fumaric acids, vindolinine, glucose, glutamate, and lactate. While the most likely metabolites observed with the aqueous fraction of stem were branch chain amino lactate, alanine, pyruvate, glutamate, malic acid, glucose, chlorogenic acids, fumaric acid, secologanin, quercetin, fumaric acids, and loganic acid, The loganic acid was only observed in the aqueous fraction of the stem.

### 3.5. Proton Nuclear Magnetic Resonance Spectroscopy

In order to perform the 1H-NMR analysis, the samples that had been extracted were mixed with D_2_O again until they reached a volume of 600 μL, which was the needed amount. Both the frozen and modified extracts were treated with 0.5 mM sodium trimethylsilyl-2,2,3,3-d4-propionate (TSP) in order to alter the chemical calibration. We prepared every single one of the samples over the course of two days, and they were then kept in storage at 4 °C. 1H-NMR A spectrometer equipped with a 5 mm broadband inverse probe was used to record spectra at MHz and 300 K. The 1H studies were conducted in one dimension using a 90° pulse angle and a little pre-saturation of the water resonance. With a capture period of s and a relaxation delay of 5 s, 512 shifts were used to collect NMR spectra. In all of these methods, a single 1H-NMR scan is all that is needed to quickly produce metabolomics data from a sample (5 s for 64–128 scans, for example). Chenomx NMR Suite 8.1 was used for processing and analysis of the spectra. To account for a line-broadening of 0.5 Hz, the spectra that had been forward-transformed were multiplied by a logarithmic weighted function. The chemical shifts of all the bands were compared to TSP, and they were all hand-corrected and adjusted for baseline. Chenomx was used to perform an analysis of the spectra that were obtained, and the specific metabolites that were found were calculated in each of the specimens. With the use of the integrated Chenomx, the Human Metabolome Database [45], and further research on plant metabolomic studies, after analysis, the metabolite-related spikes of the produced bands were assigned to the corresponding chemical shifts. The identified molecules were evaluated and validated by comparing them with the chemical shift readings obtained from additional NMR-based metabolomics investigations that were carried out in and by comparing them using the other Metabolomic databases. The identified metabolites were analyzed by Chenomx software (Chenomx NMR Suite 8.1) and with respect to the internal standard TSP. All the NMR spectral data were used to identify and characterize metabolites [46].

## 4. Conclusions

Given the rising death rate over the past decade and the increasing complexity of the disease, the need for innovative and safer therapeutic agents is urgent. Through this NMR analysis, 2,3 butanediol, quinic acids, vindoline, chlorogenic acids, vindolinine, secologanin, and quercetin are just some of the phytochemicals and terpenoid indole alkaloids (TIAs) that were produced in abundance in the leaves and less in the stem of *C. roseus*, a few of which as chlorogenic acid and vindolinine are likely to have their significant antibacterial activities. These chemicals are significantly extracted in polar solvents (water) and not in nonpolar solvents (hexane). The strong antibacterial properties of the aqueous extracts are seen in their ability to fight both Gram-positive and Gram-negative bacteria. The chemical constituent of this plant extracted in water could be an alternative antimicrobial molecule. Thus, the active chemical of this plant extracted in water needs to be explored more through in vivo studies.

## Figures and Tables

**Table 1:** The data was obtained after performing molecular docking against penicillin-binding protein 4 (dacB) from *Escherichia coli* (PDB:2EXB) and selected compounds using the AutoDock 4.0 tool.

Complex	Binding Energy	No. of Hydrogen Bonds	Residues Forming Hydrogen Bond	Residues Involved in Hydrophobic Interaction
2exb- Flomoxef	−7.1	11	SER62, ARG361, SER420,GLN422, SER357,SER306,LEU421,ASP155	PRO152,SER398,THR418, GLY419,ASN308,ALA61, GLY358,LYS65,LEU359,ASP155,LYS417
2exb- Chlorogenic Acid	−7.7	6	SER420,GLY358,SER357,SER306,	LEU421, SER62,LYS305,GLY419, THR418,LEU359,ALA61
2exb-quercetin	−7.5	3	ASN308,ARG361,LEU359	SER398,LYS417,THR418,SER420,LEU421,SER62, ALA61,GLY358,PHE160,LYS305,SER306
2exb-vindolinine	−6.9	2	ASN308,SER420	LYS65,ALA61,GLY358,LEU359,LEU421,LYS305,SER306, SER62,PHE160

**Table 2:** List of natural compounds selected for virtual screening against penicillin-binding protein 4 (dacB) from *Escherichia coli* (PDB:2EXB).

PubChem IDs	Compound Names	SMILES IDs	2D Structures
CID:262	2,3-Butanediol	CC(C(C)O)O	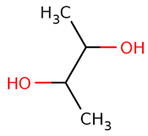
CID:6508	Quinic Acid	C1C(C(C(CC1(C(=O)O)O)O)O)O	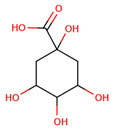
CID: 260535	Vindoline	CCC12C=CCN3C1C4(CC3)C(C(C2OC(=O)C)(C(=O)OC)O)N(C5=C4C=CC(=C5)OC)C	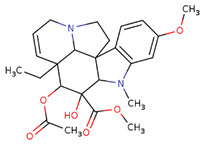
CID:525	Malic Acid	C(C(C(=O)O)O)C(=O)O	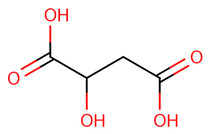
CID:1794427	Chlorogenic Acid	C1C(C(C(CC1(C(=O)O)O)OC(=O)C=CC2=CC(=C(C=C2)O)O)O)O	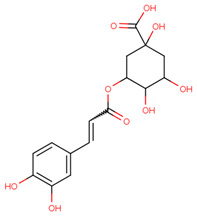
CID:444972	Fumaric Acid	C(=CC(=O)O)C(=O)O	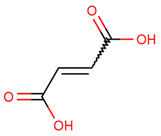
CID: 24148538	Vindolinine	CC1C23CC(C14C5(C2N(CC5)CC=C3)C6=CC=CC=C6N4)C(=O)OC	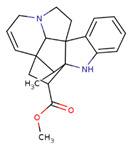
CID: 161276	Secologanin	COC(=O)C1=COC(C(C1CC=O)C=C)OC2C(C(C(C(O2)CO)O)O)O	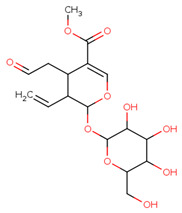
CID: 5280343	Quercetin	C1=CC(=C(C=C1C2=C(C(=O)C3=C(C=C(C=C3O2)O)O)O)O)O	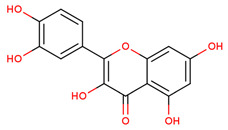

## Data Availability

Data is contained within the article.
